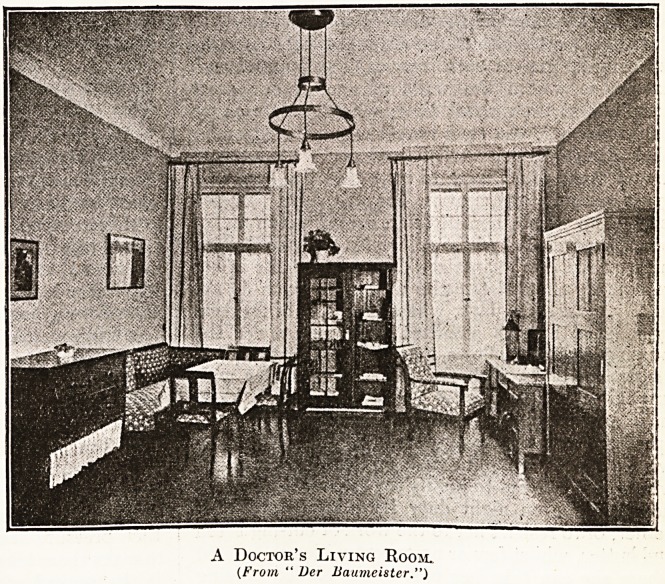# Government (Teaching) and Municipal (Non-Teaching) German Hospitals

**Published:** 1912-11-30

**Authors:** Henry Burdett


### GOVERNMENT (Teaching) and MUNICIPAL (Non-Teaching) GERMAN HOSPITALS.

By SIR HENRY BURDETT, K.C.B.

### VII. Medical Treatment and Allowances for Kasse Patients.

POINTS FOR APPROVED SOCIETIES.

At a time like the present, when the question of how to provide insured persons, being members of approved societies, with the hospital treatment they need is under discussion, it may be useful to say something of the German Kassen or approved societies. At first there were a large number of separate Kassen, or approved societies, in most of the large towns, but the fashion grew up of calling a conference of all such societies in each centre, which resulted frequently in amalgamation. Taking Leipzig as an example, we find that at first, *i.e.* in 1883, the tendency was, as the National Insurance Act permitted, to form a large number of approved societies in each centre of population. Thus there were societies for local sickness, for trade sickness, for corporate insurance, for labourers, for builders, and so forth, with many others, amounting in all, in this one town, to eighteen Kassen, in addition to a municipal sickness insurance office. After four years' experience the ruinous extravagance and undesirability of continuing this system became so apparent that the eighteen societies were united into one society, the Local Sickness Kasse for Leipzig and District, which was open to all the persons concerned within the area, without distinction of trade. This society granted to its members free medical treatment, free choice of doctors, medicines, spectacles, trusses and other appliances, to the value of 75 marks, from the first day of illness. Its objects were: (*a*) the provision of one common business and Kasse office; (*b*) a common staff and sick visitors; (*c*) the conclusion of common contracts with the doctors, chemists, and hospitals; (*d*) the provision of common cure and nursing homes for sick members. It was determined to make the most of sickness insurance, so that it should be rich in blessings to the community. With this in view the strictest centralisation to ensure equal rights and equal obligations for all insured persons, and to simplify the administration to the utmost was secured by amalgamation into one great Local Sickness Kasse for Leipzig and the District. Separate contributions for family insurance were not required, but the scale of contributions providing for an indispensable increase were fixed as follows: To end of 1903, 3 per cent. of the average daily wage; to end of 1910, 3½ per cent. of the average daily wage; and to 1911 4 per cent. of the average daily wage.

### Some of the Benefits Obtained.

In cases of incapacity Kasse sick-pay was provided up to 16½ marks per week, from the second day of illness for a period of thirty-four weeks, or as an alternative free nursing and treatment in a hospital, clinic, or convalescent home, with a cash payment to dependants of members, during the period of treatment in such an institution, equal to two-thirds of the sick pay, varying to 1s. 8d. a day. Maternity benefit, nursing-mother benefit equal to the sick pay for six weeks, a death payment of 100 marks, and for the families of members living within the area, being non-workers, in case of sickness and not themselves members of the Kasse, free medical treatment and medicines, but not appliances, for not exceeding thirteen weeks, with the right to renewal after an interval of six weeks. In the case of the death of a wife or child, being non-members, a payment of 40 marks and 20 marks respectively is made.

The Dining Room in the Medical Officers' Casino, Schwabing Hospital, Munich.
(*From "Der Baumeister."*)

### The Kasse Practitioners and Fees.

It is interesting to note that up to May 7, 1904, there existed a limited choice of doctors—that is, the selection was confined to the doctors on the Kasse list. On April 1, 1904, there existed a system of whole-time appointed Kasse doctors.

Then the doctors who had been hitherto in practice in Leipzig struck, and the commissioners took the opportunity to set aside this system, and made extensive concessions to the doctors, which, however, were only on paper. On the resumption of the temporarily suspended family treatment the doctors were compelled to reduce the fees allowed them by the authorities. Arising out of this, and the friction in working, a new general medical agreement was made at the end of 1910 between the Board of the Kasse and the Imperial Compulsory Organisation of the Doctors—that is, the medical district societies for Leipzig city and for the Leipzig rural area. Under this every doctor within the town or rural area of Leipzig has the right to be admitted as a Kasse doctor, in so far as he is willing to treat patients at the agreed fees, with other conditions, but the Kasse makes a separate agreement with each individual doctor. At the present time the doctors' fee, inclusive of the treatment of dependants, is calculated at 7¼ marks per head per annum of the average membership number.

THE DOCTORS' BOWLING ALLEY.
*(From "Der Baumeister.")*

The total medical fee is paid to a confidential committee elected by the doctors, which distributes the amount among the doctors in proportion to the extent to which they have been engaged in Kasse practice. The amount paid for an individual case is thus a fluctuating quantity, being dependent on the one hand upon the total number of cases, and on the other upon the total amount of the medical honorarium payable on the average membership figure. Beyond this inclusive fee the only extras paid for are labour cases, special electro-medical treatment, mileage, and postages. It may be interesting to give the following table, which shows the amount paid in 1911, including the fees of non-association doctors.

| | Mks. |
|---|---|
| Confinements, labour cases (to the associated doctors) ... ... ... ... | 85,657.35 |
| Honorarium to the salaried doctors ... | 23,700.00 |
| Outside Kasse doctors ... ... ... | 101,310.75 |
| Non-Kasse doctors ... ... ... ... | 41,309.03 |
| Polyclinics, etc. ... ... ... ... ... | 30,389.10 |
| Dentists ... ... ... ... ... ... | 115,071.30 |
| Confidential doctors ... ... ... ... | 22,165.00 |
| Massage ... ... ... ... ... ... | 8,301.90 |
| Zander Institute (Röntgen, etc.) ... ... | 11,634.50 |
| Supplements to doctors' fees (as per 50 and 57 of the Act) ... ... ... ... ... | 5,845.68 |
| Allowed direct to members ... ... | 10,910.18 |
| Stamping fees ... ... ... ... | 14,462.20 |
| | Mks. 470,756.99 |

The average membership in 1911 of this Kasse was 194,365 persons. We may add, as showing the working of the system, that in the first quarter of 1912 the total fees of the Kasse doctors amounted to, in round figures, 559,500 marks, but this sum is only covered by the quarter's payment of 353,387 marks—that is, to the extent of 63.14 per cent. When, therefore, the quarter's fee of a given doctor amounted to, say, 1,000 marks, he only received 63.14 per cent., or 631.4 marks.

### THE KASSE AND ITS MEDICAL DEPARTMENT.

The working of the Kasse, so far as the medical department is concerned, is conducted by the board and the Doctors' Committee. Each doctor sends in his account to the Kasse, where, after examination, it is passed on to the Doctors' Committee for further examination. If an individual doctor shows a tendency to "over-doctoring," or too great a compliance in certifying incapacity for work, or the medicines ordered are disproportionately expensive, the Doctors' Committee strikes out the fees of the doctor in question. In the case of "over-doctoring" the reduction operates to the advantage of the remainder of the associated doctors, but if it is a question of too great readiness to certify incapacity, or the prescription of too expensive medicines when cheaper ones would answer the purpose, the reduction operates in favour of the Kasse.

The Doctors' Committee exercises the following disciplinary measures: (1) Selection, (2) written warning, (3) after two warnings temporary exclusion (one to twelve months) from Kasse practice. There is an appeal from the decisions of this committee to an arbitration court, consisting of three Kasse arbitrators, three arbitrators for the local medical association, and three arbitrators representing the higher supervising authorities. The Leipzig Kasse now has contracts with 406 practitioners, including 135 specialists, and the members can choose any one of them in each case of sickness.

In case of dispute between the Kasse and the trade accident unions detailed informations have to be supplied on the request of the Kasse and all necessary documents have to be submitted. Where a practitioner exhibits a want of proper care in certifying incapacity an indemnity for the extra outlay caused thereby is deducted from the doctor's fees, the amount of such deduction being fixed by the Doctors' Committee. There is a rule that the number of Kasse doctors, including specialists, but excluding dentists, shall not sink below 250. The right of giving notice is suspended so long as the number of practising doctors falls below this figure. In case of simultaneous notice (dismissal) of doctors the right of selection belongs to the Kasse. Medicines for the Kasse are supplied by all chemists in the district at 20 per cent. discount on prescriptions and a special rebate on stock sales as agreed with the Kasse.

### THE CONTROL OF SICK MEMBERS AND FINES.

There are also three confidential doctors acting as officials of the Kasse who are responsible for the examination of the voluntary applicants before acceptance, the examination of the members notified as incapacitated by the Kasse doctors and the inspectors, the approval of the medicines prescribed, and the consideration of other technical questions. In the case of sick members in receipt of sick pay the Kasse doctor prescribes a definite morning and afternoon time, of longer or shorter duration, for outdoor exercise, or he may prohibit such exercise altogether. If the member does not conform to the rules of the doctor a fine equal to three days' sick pay for each offence may be imposed. The supervision of patients in receipt of sick pay is carried out by 25 professional and some 300 voluntary sick visitors. The Kasse area is subdivided for this purpose into sub-districts, and each of these is supervised by a foreman, whose duty it is to look after the business of his district, and to keep in close touch with the management and the whole of the sick visitors under him. The voluntary inspectors consist of members who carry out their duties in their spare time in the evening or on Sundays without any payment, but they were paid in 1911 11,963 marks for out-of-pocket expenses.

### THE KASSE'S MENTAL CONVALESCENT HOME, SANATORIA, GYMNASIUM.

This Leipzig Kasse has three sanatoria, to which it sends patients, and also possesses its own convalescent home for male nerve patients, with the right to use a sanatorium at Oberholz for female patients. Altogether the Kasse keeps occupied from 265 to 300 beds in these institutions. For the last twelve years the Kasse has owned a Zander Institute, fitted with about 100 Swedish mechanical Zander apparatus, which is under the management of two orthopædic surgeons, and of which members are granted free use. The Kasse further possesses two open-air stations where consumptives can stay during the day in the summer months. In such cases the Kasse pays in addition to full sick pay the daily fare to and from these stations, and supplies free food throughout the day.

### THE ACCUMULATION OF RESERVES.

It may be interesting to add that the German law provides that each Kasse must accumulate a reserve fund. This reserve must equal the amount of one year's expenditure calculated on the average of the last three years, and must be retained at this amount. This revenue reserve enables extraordinary expenditure, as in the case of an epidemic, to be met without an increase in the levy upon the members which must otherwise take place. For this reason the reserve has to be invested so as to provide that a good sum in cash can be readily forthcoming whenever required.

A DOCTOR'S LIVING ROOM.
*(From "Der Baumeister.")*

*(To be continued.)*

## Figures and Tables

**Figure f1:**
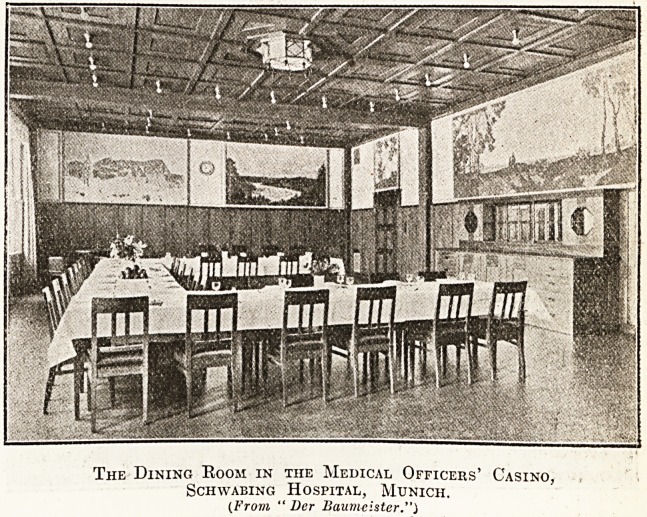


**Figure f2:**
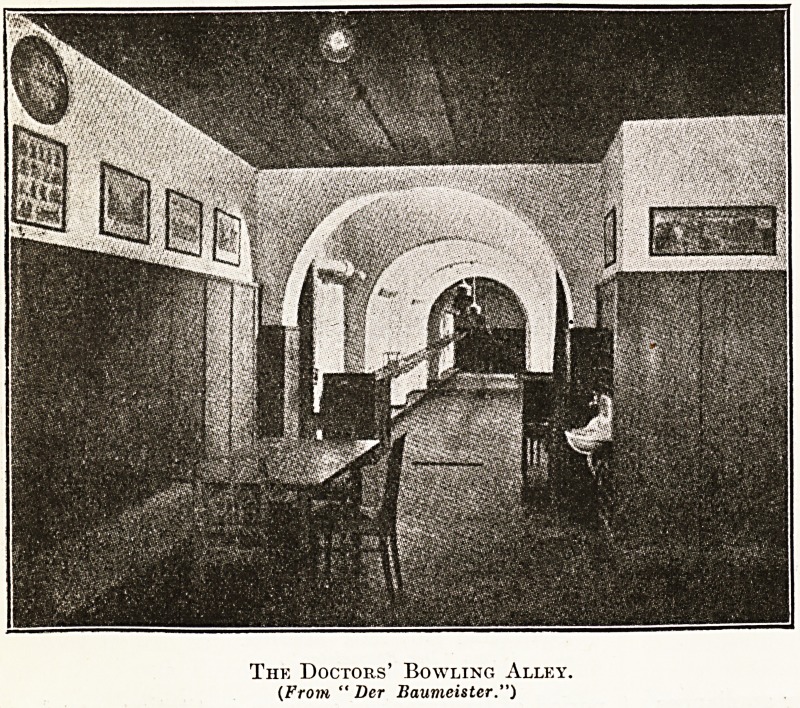


**Figure f3:**